# Comparison of Footsteps Using Connected Bracelets with the Timed Up-and-Go Test and the 6-Minutes Walking Test in a Prospective Colorectal Surgery Cohort

**DOI:** 10.3390/nu12020563

**Published:** 2020-02-21

**Authors:** Benoît Romain, David Martin, Thibaut Fabacher, Basile Pache, Dieter Hahnloser, Nicolas Demartines, Martin Hübner

**Affiliations:** 1 Department of Visceral Surgery, University Hospital CHUV and University of Lausanne, 1011 Lausanne, Switzerland; ben.romain@hotmail.fr (B.R.); david.martin@chuv.ch (D.M.); basile.pache@chuv.ch (B.P.); dieter.hahnloser@chuv.ch (D.H.); martin.hubner@chuv.ch (M.H.); 2 Groupe Méthode en Recherche Clinique, Pôle Santé Publique, Hôpitaux Universitaires de Strasbourg, 1 place de l'hôpital, 67000 Strasbourg, France; thibaut.fabacher@chru-strasbourg.fr

**Keywords:** six-minutes-walking test, timed up-and-go test, footsteps, connected bracelet

## Abstract

Preoperative physical activity and early postoperative mobilization are key components of enhanced recovery programs but both difficult to assess. The aim of this prospective study was therefore to compare different ways to measure preoperative physical activity and to correlate those tests with postoperative physical activity (footsteps). The daily number of footsteps was recorded from preoperative day 5 to postoperative day (POD) 3 in a prospective cohort of colorectal patients using connected wrist bracelets. Timed Up-and-Go Test (TUGT) and 6-Minutes Walking Test (6MWT) were assessed preoperatively. Pearson correlation and multivariable regression were used to study the predictive potential of these tests for postoperative footsteps. A total of 50 patients were included. Mean number of preoperative and postoperative footsteps were 6163 (SD 4274) and 1183 (SD 1828), respectively. There was no correlation between preoperative footsteps and preoperative tests (TUGT and 6MWT) as well as between preoperative tests (TUGT and 6MWT) and postoperative footsteps. Postoperative physical activity was significantly correlated with mean number of preoperative footsteps (Rho = 0.527, IC 95 [0.28;0.709]; *p* < 0.001). Thereby, preoperative footsteps measurement was the only tool permitting to predict postoperative footsteps. Other preoperative tests as TUGT and 6MWT could not predict immediate postoperative physical activity.

## 1. Introduction

Major abdominal surgery provokes a physiologic stress response and is associated with a period of disability [1]. One key dimension of recovery is physical activity, as it affects the ability to perform activities of daily living, return to work, and to resume social activities. Furthermore, postoperative physical activity is key to preventing potentially life-threatening complications like pneumonia, pulmonary embolism, and myocardial infarction [2]. The postsurgical period is associated with a 20% to 40% reduction in physiological and functional activity even in the absence of complications. This concerns in particular elderly patients with comorbidities who may not return to preoperative function for several months [3,4].

Intraoperative and postoperative periods were the main focus of enhanced recovery after surgery programs by fostering early postoperative mobilization. Several studies have shown the impact and the interest for intervening during the preoperative period to improve postoperative recovery [5,6]. However, preoperative and postoperative physical activities are rarely reported and difficult to quantify [6]. Further, it would be useful to preoperatively identify patients with reduced postoperative mobilization capacity. 

Several tests have been proposed to measure physical activity. The 6-minute walking test (6MWT) is based on a common daily activity and aims to measure the functional exercise performance, particularly in patients undergoing cardiac rehabilitation [7,8]. The 6MWT is often recommended to estimate functional exercise capacity [4,6,7]. Gait speed has been evaluated to assess physical performance and in particular, the Timed Up-and-Go Test (TUGT) [9]. This simple test is feasible even in a limited-resource environment to assess physical performance. Lastly, connected bracelets are an original tool measuring perioperative physical activity by the number of footsteps [10].

The aim of the study was to compare the preoperative measurement of footsteps using connected bracelets with the timed up-and-go test and the 6-minutes walking test in a prospective colorectal surgery cohort, and to correlate these preoperative test with postoperative mobilization (number of footsteps).

## 2. Materials and Methods 

This prospective study included a cohort of non-selected elective colorectal patients (except stoma closure) operated from June 2016 to September 2018 at Lausanne University Hospital (CHUV). The sample size was set at 50 patients as a pilot study. The Institutional Review Board approved the study in September 2015 (protocol number 383/15) and all patients provided written consent before surgery. The study was registered under clinicaltrials.gov in November 2015 (NCT02610790). All patients were treated according to the ERAS® protocol including routine control at 30 days after surgery [1,11].

The number of daily footsteps was recorded via connected bracelets, then stored on software and reported to a coded database. The bracelet was carried by patients from preoperative day 5 to postoperative day 4 (POD 0+1+2+3+4). Patients were instructed to wear it on the wrist of their non-dominant hand. Timed Up-and-Go Test (TUGT) and 6-Minutes Walking Test (6MWT) assessed functional capacity. TUGT was measured as the time in seconds taken to rise from a chair, walk 3 meters, turn, walk back and sit down again [9]. For 6MWT, patients were told they had 6 min to walk back and forth along the hallway at their usual pace, and the total distance was recorded in meters using the accelerometer. These tests were assessed preoperatively between one and two weeks prior to surgery through consultation with a dedicated nurse. The theoretical normal value for 6WMT depends on gender, age, height, and weight [7,12]. Following equations were used in order to calculate normal value 6-Minutes Walking Distance (6MWD) according to Enright et al. [12]: for men, 6MWD = (7.57 × height(cm)) – (5.02 × age) – (1.76 × weight(kg)) –309 m, and for women, 6MWD = (2.11 × height(cm)) – (2.29 × weight (kg)) – (5.78 × age) + 667 m. Patients were grouped as “fast” if 6MWT was higher than the theoretical value and “slow” if it was lower than the theoretical value. TUGT results were grouped as “fast” if ≤ 10 seconds, “intermediate” if 11–14 seconds, and “slow” if ≥ 15 seconds [13].

Descriptive statistics for categorical variables were reported as frequency (%), while continuous variables were reported as means (standard deviation, SD). ANOVA was used for comparison of categorical variables. All statistical tests were two-sided and a level of 0.05 was used to indicate statistical significance. A statistical correlation was measured by the use of the Pearson correlation coefficient. Data analysis was performed using SPSS 25 (SPSS Inc., Chicago, IL, USA).

## 3. Results

**Figure 1:**
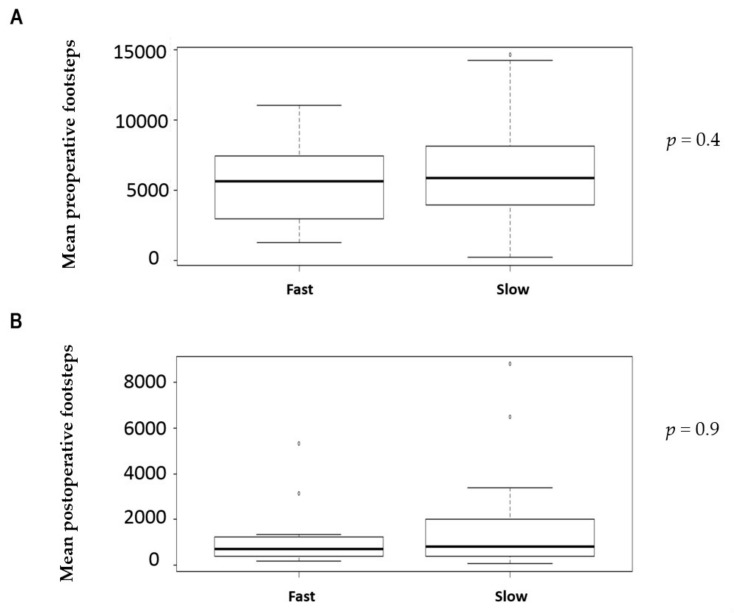
Comparison between 6-Minutes Walking Test (6MWT), preoperative, and postoperative footsteps: (**A**) Comparison between mean preoperative footsteps and 6MWT classification (slow vs fast); (**B**) comparison between mean postoperative footsteps and 6MWT classification (slow vs fast).

**Figure 2:**
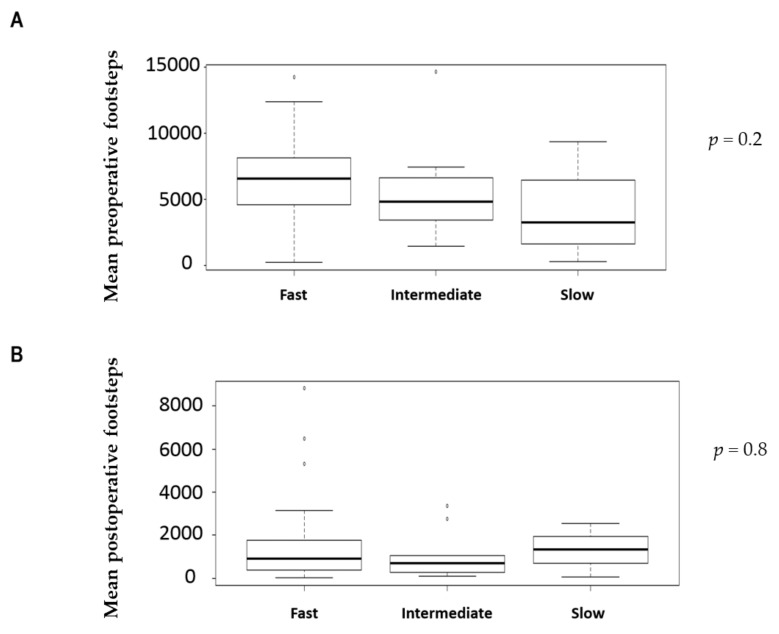
Comparison between Timed Up-and-Go Test (TUGT), preoperative, and postoperative footsteps: (**A**) Comparison between mean preoperative footsteps and TUGT classification (slow, intermediate, and fast groups) (**B**): Comparison between mean postoperative footsteps and TUGT classification (slow, intermediate, and fast groups)**.**

**Figure 3:**
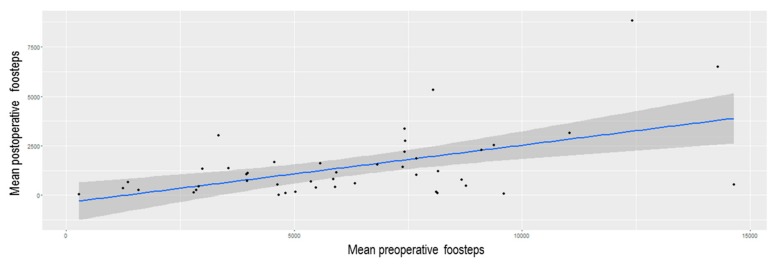
Correlation between preoperative footsteps and postoperative footsteps. r = 0.527, IC 95[0.28;0.709]; *p* < 0.001.

A total of 50 colorectal patients were included in this prospective study. Demographics and surgical details are displayed in Table 1. The mean number of preoperative and postoperative footsteps were respectively 6163 (SD4274) and 1183 (SD1828). Preoperative physical activity was correlated significantly to preoperative footsteps (*p* = 0.01), but there was no correlation between preoperative physical activity and postoperative footsteps. No correlations were found between co-morbidities, age, gender, and preoperative or postoperative functional activity. There was no significant difference between number of preoperative footsteps and 6MWT groups (slow vs fast) (Figure 1A), between preoperative footsteps and TUGT’s groups (slow, intermediate, and fast groups) (Figure 2A), just like between number of postoperative footsteps and 6MWT groups (slow vs fast) (Figure 1B). There was no significant difference between postoperative footsteps and TUGT’s groups (slow, intermediate, and fast groups) (Figure 2B). The number of postoperative footsteps was significantly correlated with number of preoperative footsteps (r = 0.527; IC 95 [0.28;0.709]; *p* < 0.001) (Figure 3). No correlations were found between active smoking, the existence of cardiovascular disease, age, and the number of preoperative and postoperative footsteps.

## 4. Discussion

Connected bracelets permitted to quantify physical activity during the perioperative course. This study showed that preoperative footsteps measurement was the only tool predicting postoperative physical activity (footsteps) during POD 0+1+2+3, whereas preoperative tests as TUGT and 6MWT could not predict preoperative nor postoperative physical activity.

Cancer is one of the major indications of colorectal surgery. In this case, fatigue and anxiety have been shown to impair both physical activity and the healing process [14,15]. Limited physical capacity was associated with poorer postoperative complications and prolonged hospital stay after elective colorectal surgery within an enhanced recovery program [16]. Prehabilitation can reduce overall and pulmonary morbidity following major surgery and could be utilized routinely [17]. Optimal prehabilitation protocols are not well defined though. One of the limits to prehabilitation and rehabilitation programs is to objectively measure physical activity. 

This quantification of physical activity could be performed with a connected bracelet which is a good tool thanks to its low price, easiness to use, and objective measurements [10]. TUGT and 6MWT are preoperative tests which are simple to set up. However, this study showed that these tests did not correlate to preoperative and postoperative physical activity. Preoperative footsteps measurement was the only tool permitting to predict postoperative footsteps. Daily measurement of physical activity with connected bracelet could permit to motivate patients to enhance their physical activity. Indeed, Carli et al. [18] have shown that there was poor adherence to a physical exercise program and that it could contribute to its lack of benefit. Many people now have bracelets and watches that measure the number of footsteps and are motivated to reach the magic recommended number of 10,000 footsteps daily. Nowadays, this represents an interesting tool and cut-offs could be used in the future to set goals for patients and guidelines. However, this number as being “good” has never been validated. The evaluation and selection of patients at risk of poor postoperative physical activity and postoperative complications could permit to target special management to these patients and increase compliance to preoperative exercises. Recently, some reviews showed that prehabilitation improved significantly functional capacity and self-reported physical activity but the impact on postoperative complications was limited [19]. Furthermore, a recent randomized trial has shown that a multimodal prehabilitation program did not affect postoperative outcomes in frail patients undergoing colorectal cancer resection [20]. Thus, even in a vulnerable population, the effects of prehabilitation and preoperative physical activity may be limited. 

Main limitations of this study are a limited study sample and its heterogeneity. Potential systematic errors arising from selection bias are imaginable, as more health-conscious and younger patients are more likely to participate in a study of this nature. The number of footsteps could also be over- or underestimated, due to the measurement uncertainties linked to the device. Postoperative complications could affect postoperative mobilization and it could be a confounding factor. This study should, therefore, be considered as a pilot study and additional prospective investigations are required to test the generated hypotheses. 

## 5. Conclusions

The number of preoperative footsteps was significantly correlated to postoperative physical activity and could be used to motivate and identify patients at risk for insufficient postoperative mobilization, while TUGT and 6MWT could not predict with reliability preoperative nor postoperative physical activity.

## Figures and Tables

**Table 1:** Patient demographics, surgical details, and physical activity.

		Overall (*n* = 50)
Age (years) (mean, SD)		58.7 (18.2)
BMI (kg/m^2^) (mean, SD)		25.4 (4.3)
Gender (M: F)		34:16
Alcohol consumption		33 (66%)
Active smoking		19 (38%)
Cardiovascular disease		19 (38%)
Chronic pulmonary disease		7 (14%)
Diabetes		6 (12%)
ASA score (I–II: III–IV)		43:7
Preoperative physical activity		
None		16 (32%)
Mild		28 (56%)
Intensive		6 (12%)
Malignancy		27 (54%)
Surgical procedure		
Colon		38 (76%)
Rectum		7 (14%)
Other		5 (10%)
Minimally invasive approach		44 (88%)
Mean number of preoperative footsteps		6163 (SD 4274)
Mean number of postoperative footsteps (from POD0 to POD3)		1183 (SD 1828)
TUGT	“fast” (≤ 10 sec)	35 (70%)
	“intermediate” (11–14 sec)	11 (22%)
	“slow” (≥ 15 sec)	4 (8%)
6MWT	Fast	12 (24%)
	Slow	35 (70%)
